# Exploring organic compound preservation through long-term in situ experiments in the Atacama desert and the relevance for Mars

**DOI:** 10.1038/s41598-025-16197-w

**Published:** 2025-08-15

**Authors:** Felix Leo Arens, Jenny Uhl, Philippe Schmitt-Kopplin, Cornelia Karger, Kai Mangelsdorf, Christof Sager, Alessandro Airo, Bernardita Valenzuela, Pedro Zamorano, Dirk Schulze-Makuch

**Affiliations:** 1 Zentrum für Astronomie und Astrophysik, Technische Universität Berlin, 10623 Berlin, Germany; 2 Research Unit Analytical Biogeochemistry, Helmholtz Zentrum München, 85764 Neuherberg, Germany; 3 Section Organic Geochemistry, GFZ Helmholtz Centre for Geosciences, 14473 Potsdam, Germany; 4 Museum für Naturkunde, Leibniz-Institut für Evolutions- und Biodiversitätsforschung, 10115 Berlin, Germany; 5 Departamento de Educación, Facultad de Educación, Universidad de Antofagasta, Antofagasta, Chile; 6 Laboratorio de Microorganismos Extremófilos, Instituto Antofagasta, Universidad de Antofagasta, Antofagasta, 1240000 Chile; 7 Departamento Biomédico, Facultad de Ciencias de la Salud, Universidad de Antofagasta, Antofagasta, 1240000 Chile; 8 Department of Experimental Limnology, Leibniz-Institute of Freshwater Ecology and Inland Fisheries, 16775 Stechlin, Germany; 9 Section Geomicrobiology, GFZ Helmholtz Centre for Geosciences, 14473 Potsdam, Germany

**Keywords:** Field experiment, Atacama desert, Biosignatures, Abiotic degradation, Oxychlorines, Biogeochemistry, Lipids, Astrobiology

## Abstract

The preservation of organic compounds under extreme environmental conditions remains a critical challenge for both terrestrial ecology applications on Earth and astrobiology. In a novel long-term field experiment over 8 months, we exposed biomolecules and a model organism to natural hyperarid conditions of the Atacama Desert, one of the best Mars analog environments. We used custom-designed sample plates for long-term exposure to simulate environmental stresses that biomolecules are exposed naturally in a hyperarid environment. The multiple stressors included extreme temperature fluctuations, associated humidity changes, and intense solar irradiation. Our field experiment complements and extends the insights obtained from previously conducted short-term laboratory experiments. To investigate biomolecule stability, we embedded adenosine triphosphate (ATP), chlorophyll-a, and the cyanobacterium *Chrooccoccidiopsis* in various Mars-relevant sediments with addition of chloride and perchlorate. Our findings, which include the rapid degradation of these biomolecules, the detection of more stable degradation products, and the identification of non-enzymatic degradation pathways, reveal the critical influence of substrate and salt types on biomolecule stability. Valuable insights into biosignature preservation under extreme terrestrial conditions and a better understanding of organic signal interpretations were gained, which will provide critical insights for future Mars missions, especially when searching for past or present life.

**Supplementary Information:**

The online version contains supplementary material available at 10.1038/s41598-025-16197-w.

## Introduction

Indigenous organic compounds have been identified on Mars through in situ measurements by the Curiosity and Perseverance rovers^1,2^ and through analyses of Martian meteorites^3^. These organics have three potential sources: (i) organic material delivered from space by comets, chondritic meteoroids, and interplanetary dust particles; (ii) non-biological formation of organic molecules underMartian conditions; and (iii) potentially indigenous biotic production. Environmental conditions on early Mars may have been similar to early Earth, where volcanic activity, hydrothermal systems, and the primordial atmosphere supported the formation of prebiotic molecules^4^ potentially leading to a vast endogeneous reservoir of organic matter. Additionally, the exogeneous source has been estimated deliver up to ~ 10^4^ g/m^2^ per year of organic matter to Mars^5^ likely including amino acids^6–8^ nucleobases^6,9^ alkylbenzenes, naphthalene, higher polycyclic aromatic hydrocarbons, carboxylic acids, and complex insoluble organic matter^7–10^.

On Earth, life produces specific organic molecules that serve as markers of past and present biological activity, commonly referred to as chemical biosignatures, which include pigments, structural biomolecules and genetic material^11^. If life ever existed on Mars, it may have produced similar chemical biosignatures, making their detection a key focus of future Mars missions^12^.

Despite the expectation that space-born infall would deposit organic compounds on Mars, the gas chromatography-mass spectrometer (GC-MS) aboard the two Viking Mars landers in 1975 detected only two organic compounds, chloromethane (CH_3_Cl) and dichloromethane (CH_2_Cl_2_), which were initially interpreted to be contamination brought to Mars by the spacecraft^13,14^. In 2008, the Phoenix Mars lander conducted the first wet chemical analysis of Martian soil, revealing ~ 1 wt% perchlorate (ClO_4_^−^)^15^ which was later also detected in the Martian meteorite EETA79001 and by the Curiosity rover at Gale Crater^16,17^. These discoveries suggested that the Viking lander’s earlier detection of chloromethane (CH_3_Cl) and dichloromethane (CH_2_Cl_2_) might have been due to the exposure of indigenous organics to perchlorate^18^. Later missions have confirmed the presence of indigenous organic compounds on Mars such as the Curiosity^1,19^ and Perseverance rovers^2^ as well as the discovery of diverse organics in a number of Martian meteorites on Earth^3,20^. The discovery of perchlorate in Martian soil suggests that organic compounds might be oxidized by oxychlorines and oxygen species during perchlorate formation and its radiolytic breakdown, complicating the detection of organics^21–23^. The core issue is to understand the processes that influence the preservation, alteration, and detection of organic compounds on Mars, which is crucial for identifying potential Martian chemical biosignatures.

The hyperarid core of the Atacama Desert in Chile is often regarded as an environmental analog to Mars, given its extreme dryness, similar surface structure and salt accumulation, high UV radiation, and minimal organic content^24–26^. Previous field investigations have informed astrobiologists about life’s strategies in such extremely arid environments and how this pertains to life-detection missions on Mars^24,27–32^. In this Martian analog environment, a variety of biomolecules like lipids, carotenoids, and chlorophyll have been detected in sediments and evaporites^33,34^ providing a valuable natural setting for investigating biosignature preservation and degradation. While laboratory experiments studying biomarker stability under Mars-like conditions^23,35–41^ are usually very short, Mars analog environments like the Atacama Desert provide the opportunity to conduct long-term in situ experiments. However, these long-term experiments are rare^42^. Such field experiments not only complement laboratory investigations but also serve as valuable counterparts to space-based studies conducted aboard the International Space Station (ISS)^12,43,44^.

**Fig. 1:**
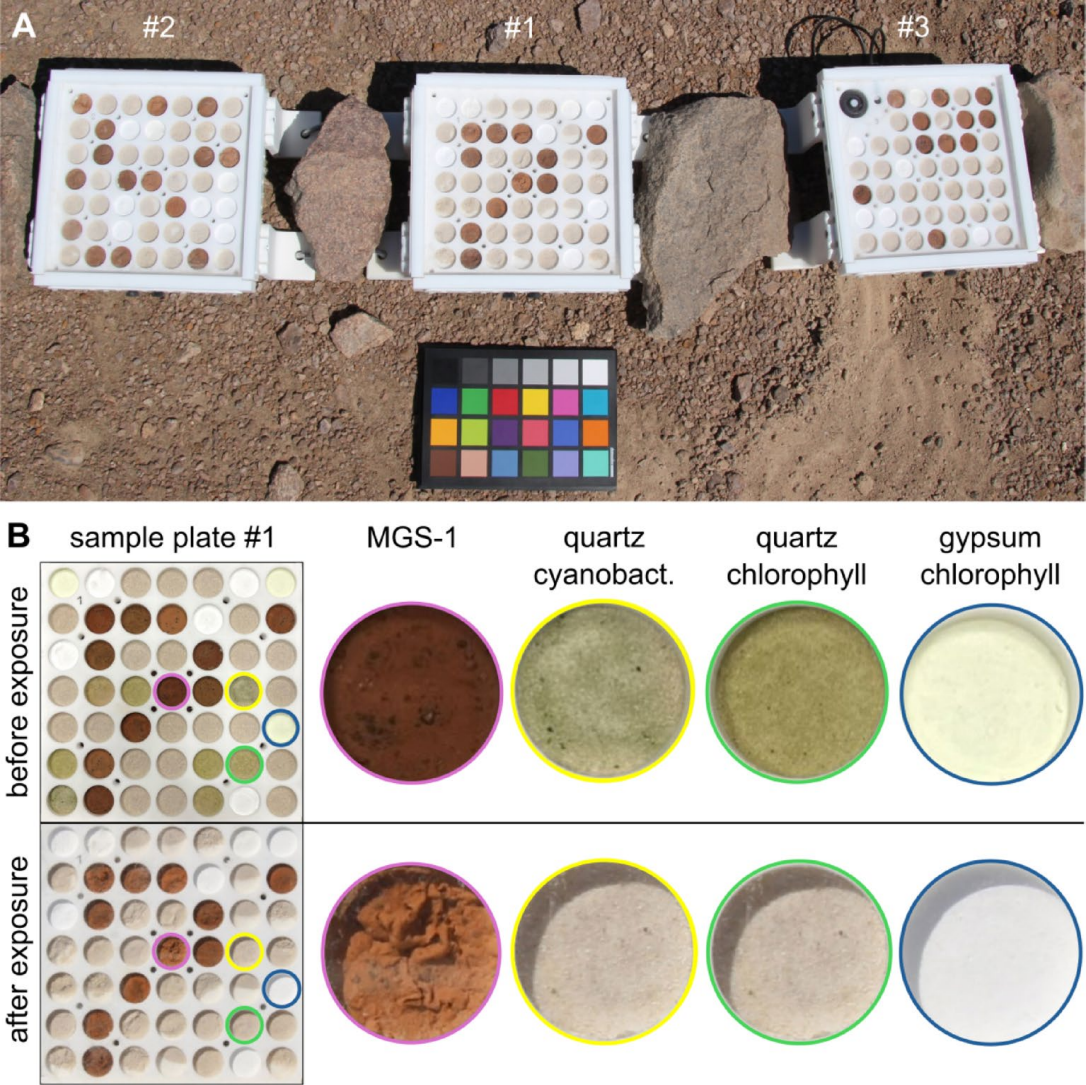
(**A**) Exposure experiment in the Atacama Desert 2 months after setup (Plate #1 was exposed 2 months, plate #2 4 months, and plate #3 8 months, an additional plate remained as a control unexposed in the lab). In the upper left corner of plate #3, the sensors for environmental monitoring are built in. Color reference card (20 × 15 cm) placed below as reference. (**B**) Sample plate #1 before (upper row) and after 2 months exposure (lower row): Examples of visible alteration are taken from MGS-1, which shows morphological deformation; the cyanobacterial samples and chlorophyll samples (in quartz and gypsum substrate) which had before exposure a greenish color has bleached completely.

To address this gap, we conducted a long-term exposure experiment in the Atacama Desert. In preparation, we built four custom-made sample plates with integrated quartz glass cover providing the ability to expose samples to the environmental conditions in the hyperarid core of Atacama Desert, while protecting them from eolian erosion and microbial contamination (Fig. 1A). Samples consisted of either ATP, chlorophyll-a, or cyanobacteria (*Chrooccoccidiopsis sp. 029*) in different Mars-relevant substrates (quartz, gypsum, and MGS-1 Mars regolith simulant^45^ (Table S 1)) and additionally 1 wt% chloride (NaCl) or 1 wt% perchlorate (NaClO_4_). We deployed three plates in the hyperarid core of the Atacama Desert (24°S 70°W), 45 km inland and 1206 m above sea level, unaffected by the coastal fog (< 1200 m elevation) for 2, 4, and 8 months. In-built sensors constantly monitored environmental parameters including temperature, relative humidity (RH), and solar influx. We handled and transported the control plate identically but kept it under laboratory conditions. The overarching goal of this field study was to understand how these organic compounds behave in the Atacama Desert, to gain insights into the processes influencing biosignature preservation in terrestrial environments with prolonged hyperarid conditions, like Mars. The study specifically focuses on the role of mineral substrates, including Mars relevant salts, in influencing the degradation of chemical biosignatures.

## Results

### Diurnal and seasonal variations during the experiment

The experiment design proved to be stable, withstanding the strong winds in the Atacama Desert while ensuring UV transmission and air circulation through filter-protected vents (Figure S 1).

Seasonal variations were subtle, though solar irradiance decreased from a daily average of 350 W/m² or higher in the austral summer to 150–200 W/m2 during the winter (Figure S 2 A). The daily solar flux closely followed the sun’s position, with only minimal cloud coverage observed. Over time, dust accumulated on the glass plate, particularly along its edges and corners, where minor crusts eventually formed. Based on measurements from the solar radiation sensor, the three exposure periods lasted 59, 120, and 242 days, resulting in solar radiation budgets of approximately 0.65, 1.19, and 1.89 kWh per sample, respectively.

During the diurnal cycle, both temperature and RH exhibited pronounced fluctuations. At night, the temperature dropped to approximately 0 °C, with RH approaching 100%, whereas during the day, the temperature peaked at about 50 °C and RH fell to around 15% (Figure S 2B). The measured temperature and RH values inside the plate are similar to the outside surface values (Figure S 3). Inside the plate, RH was slightly higher (up to 5%) during the night and morning hours, and the temperature was higher (up to 5 °C) during the day. On several nights, the temperature nearly met the dew point (Figure S 2B), enabling dew formation, which was observed on site on the morning of the 31st of January 2023 (Figure S 1).

Morphological changes of the samples were primarily dependent on the substrate type. The surface of the gypsum samples remained unchanged as a compact pellet. The grains of the quartz sand were overall the least cohesive, forming a loose sediment. MGS-1 sample surface underwent considerable alteration, forming fluffy surfaces (Fig. 1B). The addition of salt and biomolecules also influenced morphological stability: samples containing cyanobacteria showed higherstability, whereas those with salts, ATP, and chlorophyll-a were less stable. Notably, the green color of the cyanobacteria and chlorophyll-a disappeared in all exposed plates within two months (Fig. 1B).

### Rapid ATP degradation

**Fig. 2:**
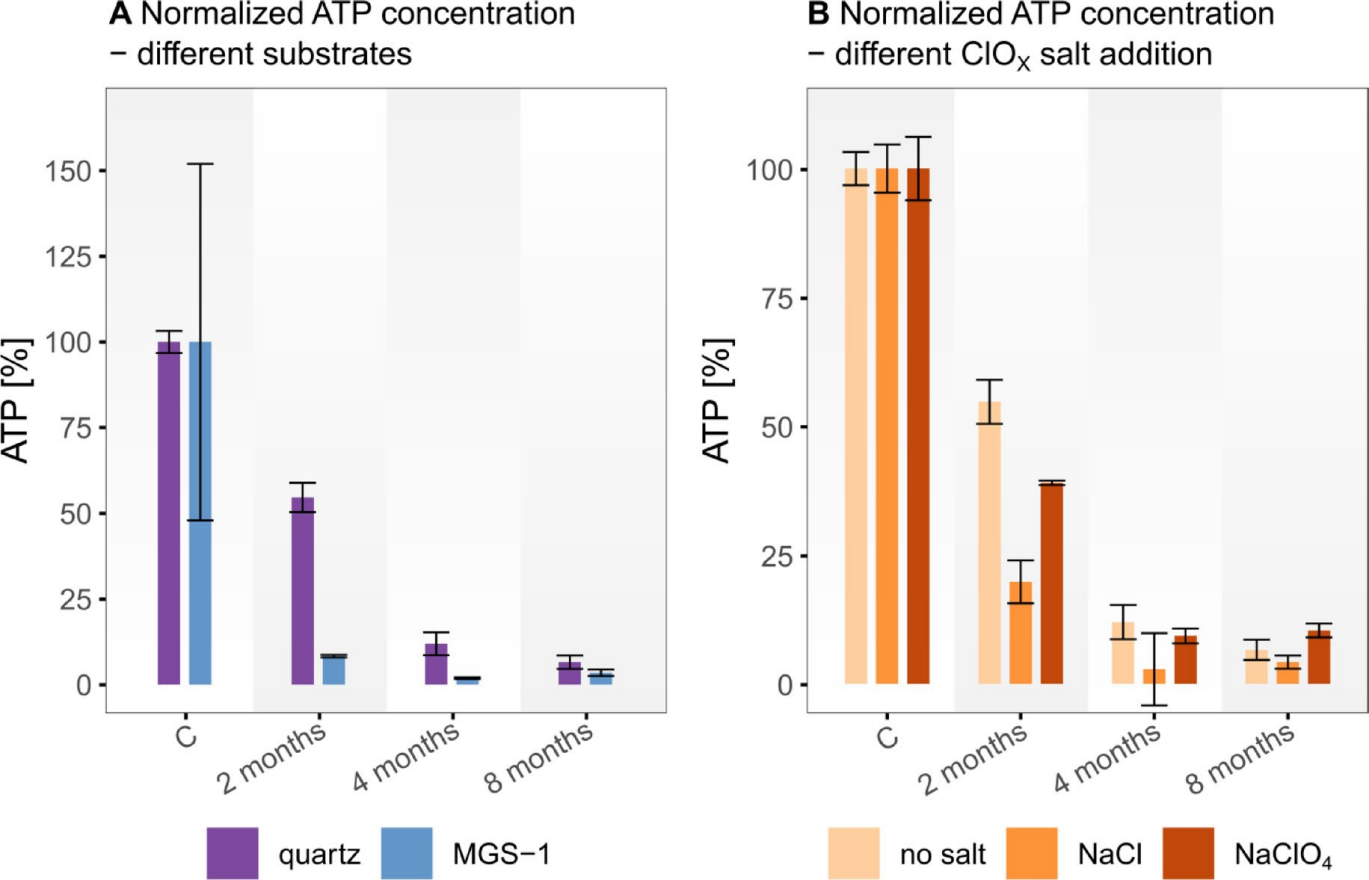
ATP concentration results of the exposure experiment after different exposure durations given in months, C = control samples without exposure. (**A **) Comparison between different substrates; (**B**) Comparison between different additions of ClOx salts. Data is normalized in relation to the control sample concentrations. The error bars represent the standard error derived from the standard addition calibration using error propagation of the linear regression parameters.

The ATP was quantified by bioluminescence via luciferin–luciferase reaction, as described in the methods. The measured ATP concentrations after different exposure durations are intriguing, showing salt- and substrate-specific degradation rates (Fig. 2, Table S 2). Although we analyzed the samples by using standard addition method, dissolved ions, leached with the ATP during sample preparation, still appear to complicate the quantification of ATP resulting in larger uncertainty. This quenching effect is most prominent in the gypsum samples with elevated concentration of dissolved CaSO_4_, which remain very difficult to analyze^46^. Here the ATP does not show a trend of degradation (Table S 2). In the other sample configurations, the ATP concentration gradually decreases (Fig. 2).

### Chlorophyll-a and its degradation products

**Fig. 3:**
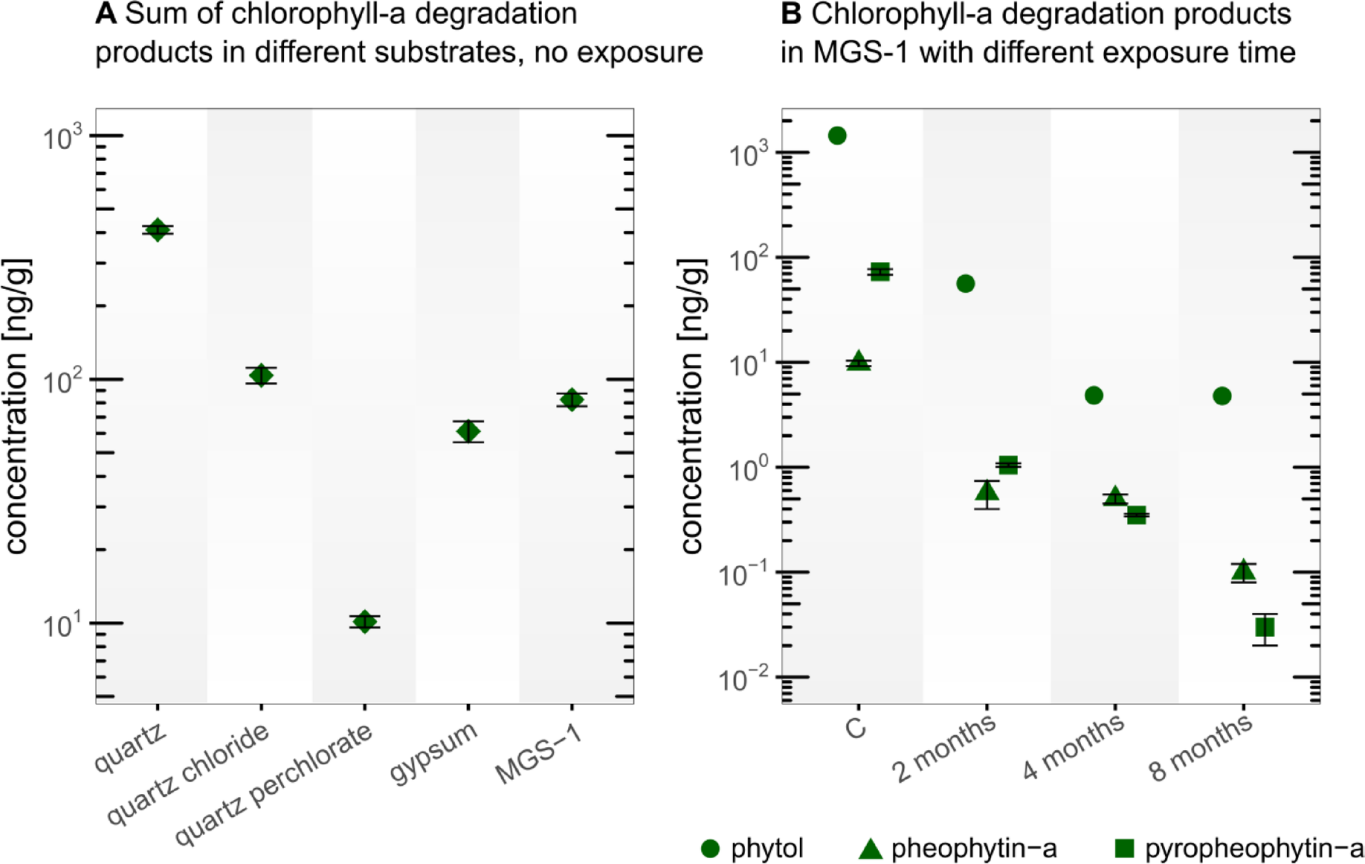
Chlorophyll-a degradation products, pheophytin-a, pyropheophytin-a and phytol. (**A**) Summed concentrations of the larger degradation molecules of chlorophyll-a (pheophytin-a and pyropheophytin-a) in the control samples of the different sediment substrates (in the exposure samples chlorophyll-a and its degradation products pheophytin-a and pyropheophytin-a decayed so fast that none could be detected except in the MGS-1 substrate). (**B**) Concentrations of all detected chlorophyll-a degradation products in the MGS-1 substrate after different exposure durations. “C” represents control samples without exposure. The error bars for pheophytin-a and pyropheophytin-a represent the ±1 SD standard deviation of experimental triplicates.

The chlorophyll-a decayed faster than expected and thus was not detected in any sample, not even in the control samples (Table S 3). However, we were able to detect thermal decay products such as pheophytin-a (loss of central magnesium ion) and pyropheophytin-a^47^ (additional loss of a methyl formate side group) in the control samples and in MGS-1 substrate over time (Fig. 3). The smaller breakdown product phytol (chlorophyll-a side chain) was detected most abundantly in all control samples and in all substrates exposed for up to 2 months except for the quartz-perchlorate substrate.

Comparing the abundance of the initial chlorophyll-a degradation products (pheophytin-a and pyropheophytin-a) in the control samples of the different substrates, it can be observed that the degradation was least advanced in the pure quartz sample followed by quartz with chloride, MGS-1, gypsum and most advanced in quartz with perchlorate (Fig. 3A). In the time series, where the substrates were exposed to Atacama Desert conditions, chlorophyll-a and its decay products, except phytol, were absent in all quartz substrates and in the gypsum substrate already after the first 2 months. Only in the MGS-1 Mars soil simulant the decay products of chlorophyll-a were detectable (Fig. 3B) over the entire 8 months of the experiment, while their abundance gradually decreased over time.

### Gradual breakdown of cyanobacteria

**Fig. 4:**
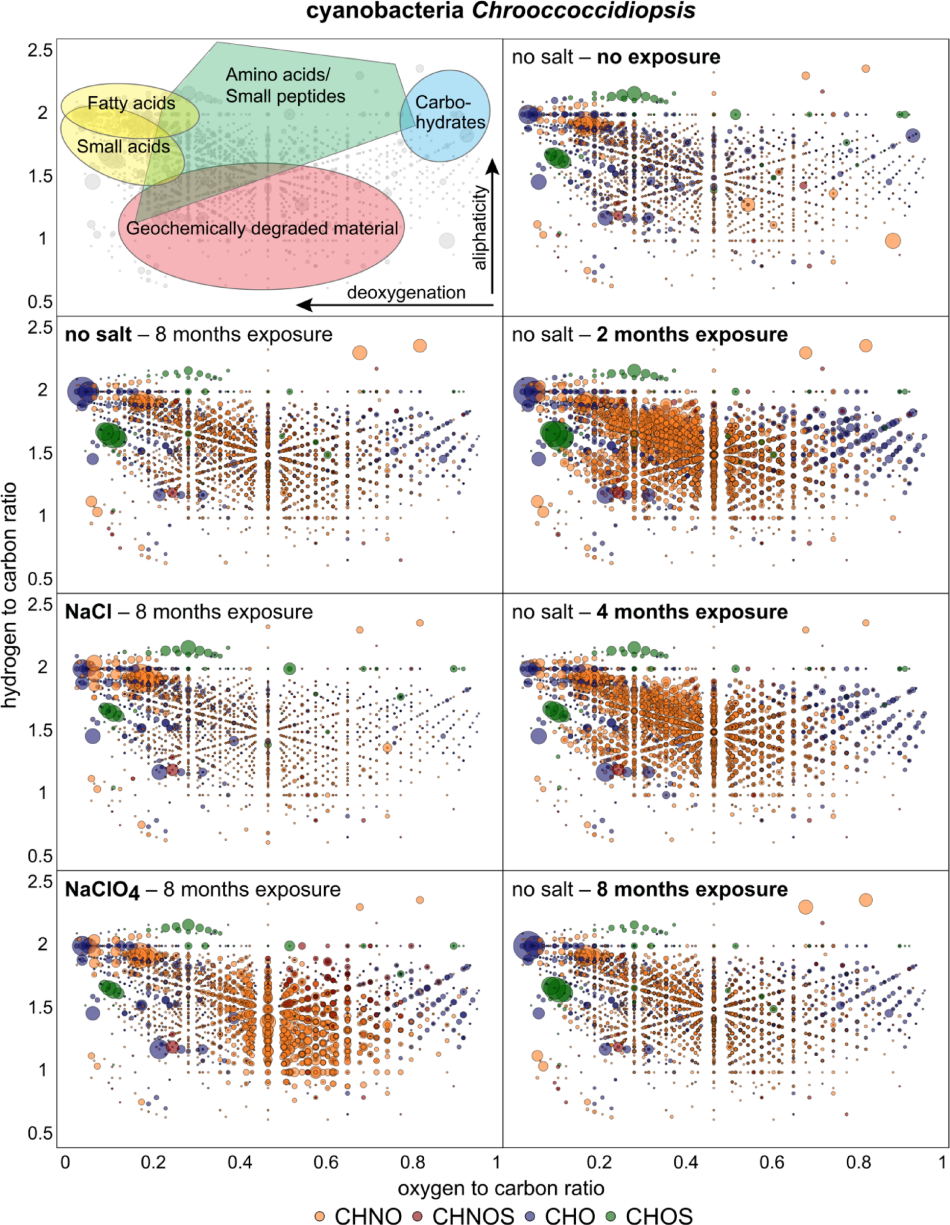
Compositional profiles of organic matter from the cyanobacterial strain Chrooccoccidiopsis remain after exposure durations. The assigned molecule masses are shown in van Krevelen diagrams plotting the hydrogen to carbon atomic ratio (H/C) as a function of the oxygen to carbon (O/C) atomic ratio of organic compounds. The upper left plot shows the positions of chemical classes (colored areas) depicted in compositional space according to their characteristic H/C and O/C ratios. Highly aliphatic compounds are mostly presented in the upper (H/C ratio > 1) and aromatic compounds in the lower area (H/C ratio < 1). The different bubble sizes represent the intensity of the characteristic molecular formula within the respective sample. Left column: cyanobacterial samples in quartz substrate after 8 months without salt, 1 wt% NaCl, and 1 wt% NaClO_4_. Right column: cyanobacterial samples in quartz substrate without salt addition after different exposure durations, including the control sample with no exposure to the Atacama Desert.

The samples spiked with a handmade cultivated cyanobacterial strain *Chrooccoccidiopsis sp. 029 (Culture Collection of Microbes from Extreme Environments [CCMEE])* were analyzed with FT-ICR-MS to monitore the degradation of the model organisms under Mars-like environmental conditions. Each mass signal was assigned to its corresponding molecular composition and classified as CHO, CHOS, or CHNO species^29^. The quartz substrate sample was analyzed with different exposure rates and spiked with NaCl and NaClO_4_. A characteristic metabolite pattern could be observed for each condition which is shown in Fig. 4. The control sample is dominated by CHO compounds, whereas the exposed samples are dominated by the appearance of nitrogen containing compounds (CHNO) during all time of exposure. The CHNO signals were most abundant in the quartz samples after 2 months of exposure and decreased with time. After 8 months the NaCl and NaClO_4_ containing samples show very distinct patterns; While the sample with NaCl is very close in nitrogen compound abundance to the no-salt sample, the NaClO_4_ sample shows a significant increased amount of more oxygenated CHNO reflecting sequestration of nitrogen compounds.

## Discussion

Our study provides valuable insights into the stability of organic compounds through an exposure experiment conducted in the Mars-analog environment of the Atacama Desert. Among the monitored environmental factors, UV radiation significantly drives biomolecule degradation^12,48,49^. Substrates like the Mars regolith simulant MGS-1 provide effective shielding compared to translucent quartz and gypsum^39^. Fe- and Mg-rich mineral phases such as olivine, and pyroxene of the MGS-1 absorb and scatter UV-light^40^ reducing photolysis rates by > 90% relative to unshielded organics^39^. These findings highlight the importance of UV‑shielding substrates for preserving organics in both the Atacama Desert and on Mars^36^.

Despite the extreme aridity, transient moisture events with RH close to 100%, evidenced by dew-induced dust films and blooming (efflorescence) in hygroscopic minerals, particularly epsomite (MgSO_4_·7H_2_O), which accounts for 4 wt% of MGS-1 ^45^^50^. The temperature and RH data indicates that for roughly 320 h throughout the experiment conditions were favorable for condensation (Figure S 4), potentially facilitating chemical reactions and biomolecule degradation^8,49^. We cannot fully exclude slight differences in the microclimate within the covered plates. However, temperature and RH data (Figure S3) show only minor deviations from ambient conditions, suggesting that exchange through the mesh-covered ventilation inlets with the environment was sufficient. Together with the harsh UV radiation, these environmental factors probably contributed to the rapid loss of organic matter observed in our samples.

Deciphering degradation pathways under Mars‑like conditions is key to assessing the preservation potential of organic signatures. For ATP, both UV radiation and heat appear to influence stability differently across substrates^38,51^. On one hand the mineral mixture of MGS-1 shields off the UV-radiation^45^. On the other hand, due to the opaque and darker minerals, the MGS-1 is more likely to heat up more rapidly and more intensely than quartz or gypsum with a high reflectivity (albedo). This could explain the higher preservation potential for ATP in the exposed quartz samples compared to the exposed MGS-1 samples (Fig. 2A). The presence of salts, particularly chloride (Cl^−^) and perchlorate (ClO_4_^−^), significantly affected ATP stability^23^. Interestingly, samples containing sodium perchlorate (NaClO_4_) exhibited higher ATP values after exposure compared to those without added salts or with other salts. This paradoxical observation may be attributed to perchlorate’s potential to inhibit microbial activity or enzymatic degradation pathways, possibly stabilizing ATP under certain conditions. More puzzling are the gypsum results, where ATP increased with exposure. Considering the environmental conditions (i.e., dew formation for considerable timespans), microbial activity of some kind or contamination via eolian input seems unlikely but possible. However, due to methodological uncertainties associated with the luciferase assay in high-salt environments, specifically ion interference and quenching effects^46^ these results should be interpreted with caution. Further research is needed to clarify the mechanisms behind this observation.

**Fig. 5:**
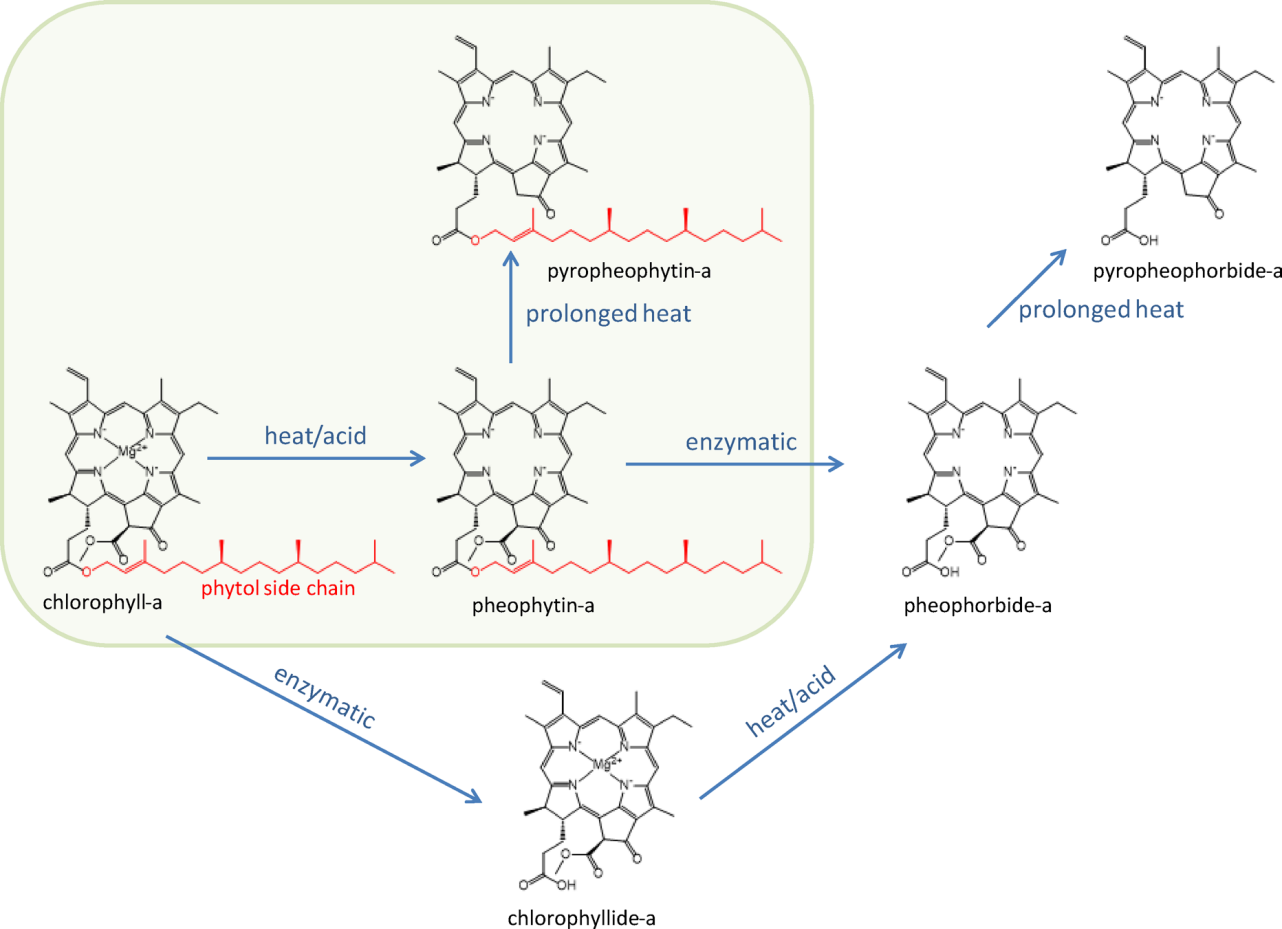
Thermal and enzymatic degradation pathway of chlorophyll-a (modified after Delpino-Rius et al., 2018). The frame indicates the heat-stimulated degradation of chlorophyll-a to the degradation products detected in this study.

Chlorophyll-a degraded so rapidly that it was not detected in any sample. Even its direct degradation products were detected only in the MGS-1, although throughout the entire 8 months of the experiment (Fig. 3, Table S 3). The detected degradation products such as pheophytin-a and pyropheophytin-a followed non-biological pathways (Fig. 5), suggesting that in the absence of biological activity, chlorophyll-a undergoes abiotic degradation^47^. This substrate-dependent detection, where chlorophyll-a breakdown products were found in the darker and opaque MGS-1 but not in the bright and translucent quartz and gypsum, strongly suggests that UV-radiation, rather than heat, is the primary driving force for degradation. Phytol concentrations followed a similar pattern, though it appeared more resistant to degradation, remaining detectable also in quartz, quartz with NaCl and gypsum substrate after 2 months of exposure. Although phytol is described as the enzymatic degradation product of chlorophyll-a (loss of phytol side chain) to chlorophyllide-a or of pheophytin-a to pheophorbide-a^34^ (Fig. 5), its enzymatic counter molecules chlorophyllide-a and pheophorbide-a were not detected. This suggests that phytol might also be an intermediate product in the final abiotic degradation of chlorophyll-a. The absence of phytol in perchlorate-containing samples (Table S 3) indicates an additional stress on the molecular structure, consistent with perchlorate’s known ability to disrupt biological molecules^52^. The enhanced preservation of these degradation products in MGS-1 underscores the crucial role of substrate composition, particularly its UV shielding, in preserving degradation intermediates. Targeting more stable degradation products like porphyrins (degradation product of chlorophyll-a) and isoprenoid hydrocarbons such as pristane and phytane (degradation products of phytol) could be a promising approach for detecting evidence of past life^53^. In previous experiments straight-chain hydrocarbons with 10, 11, and 12 carbon atoms were detected in Martian rocks conducted by the Sample Analysis at Mars instrument onboard the Curiosity rover, but an abiotic or biotic origin remains uncertain. In this context, isoprenoids might be ideal targets since they are also very stable, can be as easily detected as n-alkanes, and their more complex structural characteristics may be more indicative of a biotic source^19^. The influence of salts on chlorophyll-a degradation pathways could only be examined in the unexposed lab control samples. This is because the exposed samples, which contained salts, were mixed in translucent quartz substrates. Due to the inadequate UV-shielding, the degradation was so rapid that no chlorophyll-a or its breakdown products were detectable after just two months, except for phytol was found in the quartz-NaCl sample after 2 months exposure. However, comparing the chlorophyll-a samples in the unexposed control plate indicates a salt type dependence on the stability of chlorophyll-a and its degradation products. The clear sequence of degradation intensity without salt < NaCl < NaClO_4_ can be explained by the hygroscopic nature of the salts, where sodium perchlorate is more hygroscopic than sodium chloride. Under humid conditions, degradation of organic molecules could thus accelerate^36^. Additionally, NaClO_4_ is a chaotropic agent that disrupts hydrogen bonding thereby destabilizing structured organic molecules^49^.

FT-ICR-MS analysis revealed that the amount of organic molecules detection increased during the first four months of exposure (Fig. 4), indicating a release of intracellular compounds as cyanobacterial cells began to degrade. Environmental stresses such as UV radiation and heat cause molecular oxidation, membrane damage and cell lysis. The loss of cellular integrity led to the liberation of organic constituents into the surrounding substrate. After 8 months, however, the diversity of detected organic molecules decreased, suggesting further degradation and potential oxidation and mineralization of the released compounds (Fig. 4). Environmental factors like UV radiation, moisture fluctuations, and salt interactions significantly contributed to thesedegradation vs. conservation dynamics. Microbial contamination cannot be fully excluded but is unlikely to be significant as the chlorophyll experiments did not indicate any enzymatic breakdown products (Fig. 5). The presence of salts appeared to have the greatest effect on the cell compounds. Besides osmotic stress, UV-induced photolysis is enhanced by the presence of sodium chloride (NaCl) and sodium perchlorate (NaClO_4_). Chloride and perchlorate ions participate in photooxidation reactions under UV irradiation, leading to the formation of reactive chlorine species and for perchlorate also reactive oxygen species that accelerate the degradation of organic molecules^54,55^. As a result, nitrogen containing compounds became relatively concentrated in the NaClO_4_ exposed samples after 8 months. This relative enrichment may be explained by the higher chemical stability of many CHON compounds—such as peptides or amide-bearing molecules—whose resonance structures and reduced susceptibility to oxidation make them more resistant to ROS and RCS-mediated degradation compared to more reactive CHO, CHOS, or CHONS species. Under these conditions, nitrogen containing compounds may be promising candidates for preserved organic molecules^34,56^. The observed differences between the chloride and perchlorate samples may indicate that the production of reactive oxygen species and oxychlorines from chloride is more efficient than from perchlorate, at least under the conditions present in the Atacama Desert. On Mars this could be different, as strong cosmic radiation, responsible for the radiolysis of perchlorates, is not shielded by a magnetic field and thick atmosphere as it is on Earth^22^.

Hyperarid deserts, characterized by minimal precipitation, and subject to intense solar radiation, as well as pronounced diurnal temperature fluctuations, serve as natural laboratories for exploring the processes that govern organic molecule stability at Earth’s surface under largely abiotic conditions^29,34,57^. The rapid degradation of chlorophyll-a and ATP documented in our field experiments underscores how swiftly labile organic compounds can be transformed or destroyed under these extreme conditions. By conducting long-term in situ experiments, we bridge the gap between short-term laboratory simulations and real-world environmental dynamics. This approach enables us to investigate the threshold conditions under which chemical biomarkers either persist or degrade, thereby expanding our understanding of how hyperarid ecosystems support, or limit, organic preservation.

Our observations regarding salt composition and substrate type reflect broader findings from hyperarid regions. For instance, clay-rich subsurface layers have been associated with the preservation of organic matter over millennial timescales, with some studies suggesting stability for multi-million years^34^. This preservation is attributed to long-term hyperaridity, which suppresses both microbial and enzymatic activity and shields off from surface erosion and photochemical induced alteration. Previous field studies conducted in the Yungay Valley, where the experiment was conducted^25,28–30^highlight the harsh conditions encountered near the surface, but questions remain about how these factors specifically influence long-term stability of organic matter. Our results help address this gap by demonstrating how extended, controlled experiments provide insights, unattainable through shorter laboratory simulations.

By quantifying biomolecule degradation and preservation on different mineral and salt surfaces, this work clarifies the interplay among temperature fluctuations, UV flux, and salt chemistry, factors that collectively drive or hinder organic decay^58^. These findings have far-reaching implications for areas such as desert ecology, microbial biogeography, and paleoclimatology, where knowledge of organic stability informs interpretations of ecosystem functioning, nutrient cycling, and climatic history.

In a Martian context, our results imply that surface-exposed biomolecules like ATP and chlorophyll-a are unlikely to persist, whereas more stable organic molecules, such as porphyrins or lipids, may serve as better chemical biomarker candidates^53,56^. The cyanobacterial experiments support this, showing that fatty acids and molecules with low H/C and low O/C, like porphyrins, are among the compounds most resistant to degradation (Fig. 4). Moreover, perchlorate salts, which are also abundant on Mars^34,56^appear to favor the preservation of nitrogenous compounds in our experiment. The Mars experiments conducted during the BIOMEX mission on the ISS, which also investigated biomolecules and cyanobacterial cells under a Mars-like conditions, show results that are broadly consistent with ours^12,43,44^. Similar to our findings, compounds mixed with Mars analog minerals exhibited increased resistance to radiation. However, the organic compounds in our experiment degraded more rapidly, likely due to higher temperature fluctuations, higher water activity, and atmospheric composition, which warrant more extended field experiments. Notably, even during the Viking and Phoenix lander missions frost and dew were observed, indicating that transient hydration events can occur even in Mars’s harsh environment, indicating that even on today’s Mars water might play a role in biomolecule degradation^59,60^. Complementary to our degradation-focused findings, a recent study demonstrated that stress-adapted fungi like *Rhinocladiella similis* can produce perchlorate-induced peptides and osmolyte-like molecules, which may serve as chemical biosignature candidates under Martian conditions^61^.

The data presented here highlights the subsurface dependent shielding potentials of organic compounds in Martian environments. Substrates like the MGS-1 simulant that provide enhanced UV protection demonstrate enhanced preservation of organic compounds. This supports the ongoing effort to prioritize future missions to explore the subsurface or regions where protective substrates may preserve biomolecules. Considering the timescale of billions of years and the cosmic radiation even deeper subsurface down to 1.5 m depth should be considered, as it is proposed by the ExoMars mission^62^. Investigating such environments increases the chances of detecting signs of past or present life by minimizing the exposure to harsh surface conditions. However, the salt composition and concentration of the substrate is essential as well, as it can influence the stability potential organic matter and microbes directly and also its local microenvironment^23,35^which is also shown by our study. Besides the beneficial effects like lowering the freezing temperature and attracting water vapor, salts, and especially oxychlorines also can lead to increased degradation of organic compounds, possibly even in the subsurface^23,36^.

Despite certain limitations of the analogy between the Atacama Desert and Mars, such as differences in including water availability, atmospheric pressure and composition, this study provides valuable insights from a long-term Mars-analog field experiment. These experiments offer a link between limited Mars observations and controlled laboratory simulations. Future research should build on this approach by expanding the range of biomolecules and Mars-relevant salts, such as CaCl_2_, NaClO_3_, and NaNO_3_ to refine our understanding of organic stability under extreme conditions like on Mars^28,35,61,63^.

## Methods

### Experimental set-up

The custom-designed sample plates were developed for this specific experiment. The set-up ensured that the samples were exposed to the environmental conditions (daily fluctuating temperature, RH, and solar irradiation) while being protected from biological contamination. As base a UV- and heat-resistant PTFE platform was used. At its sides, vents allowed air to circulate between the chamber inside and outside, which were protected with 20 μm mesh filter. A 3 mm-thick glass cover protected the samples from wind erosion and cross-contamination while maintaining good UV light transmission (Figure S 5). 3D-printed legs positioned the plates 5 cm above the ground, to ensure consistent environmental exposure (e.g., air circulation, heating). Each sample plate had a total number of 49 sample pods except for the sample plate exposed for 8 months. This included the environmental sensors and therefore could accommodate only 45 sample pods (Fig. 1). Each pod measures 2 cm in diameter and 0.5 cm in depth. The samples were randomly positioned on the plates to ensure an unbiased distribution, minimizing potential influences from their location relative to the plate edges. Factors such as light exposure, gas exchange, and water vapor dynamics could affect biomolecule degradation, making randomization essential for reducing positional biases. The sample plates were prepared and equipped with the samples and the sensors under sterile conditions in the laboratory of the Technical University of Berlin. For transportation, the glass plate was exchanged with an aluminum plate, sealing off each pod. Prior to sample preparation, the sample plate and all used tools were cleaned with ethanol to prevent contamination. The following substrates were carefully cleaned and sterilized with 100% ethanol: fine quartz sand (0.1–0.4 mm grain size), gypsum powder (CaSO_4_ ∙ 2H_2_O), MGS-1 Mars simulant^45^. Samples were prepared using 0.5 g of substrate, 200 µL of salt solution, 200 µL of biomolecule solution per sample. All chemicals used in this experiment were of high purity grade. Salt solutions were prepared with 8.06 g/L NaCl and 6.1 g/L NaClO_4_ each to achieve a 1 wt% Cl^−^ and 1 wt% ClO_4_^−^ concentration. A 5000 ppm concentration for ATP and chlorophyll-a was aimed. Therefore, solutions from biochemical standards were freshly prepared. For the cyanobacterial samples a *Chrooccoccidiopsis sp. 029* strain was used, which was cultivated in a liquid BG11 medium. The cell density of 5 10^3^ cells/µL was determined with a Neubauer cell counting after vortexing the inoculated medium, resulting in 10^6^ cells per sample. The filled sample plates were mildly desiccated overnight in a desiccator (no vacuum, desiccant agent: phosphorous pentoxide) and later sealed with an aluminum plate.

### Environmental monitoring

The daily fluctuating temperature, RH, and solar irradiation were monitored with a Hobo USB Mirco Station (H21-USB) data logger (Onset Computer Corporation, USA) equipped with a silicon pyranometer light sensor (S-LIB-M003) and a combined temperature and RH sensor (S-THC-M008). The recording interval was 5 min. Both sensors were built in the sample plate with the longest exposure (8 months) and monitored the temperature and RH during the transport from the lab to the field site and back.

### ATP analysis

Samples (0.5 g) were leached in 1 mL of sodium phosphate buffer (0.12 M Na_2_HPO_4_, NaH_2_PO_4_, pH = 8.0). Samples were shaken on an orbital shaker for 10 min at 150 rpm. The samples were then centrifuged at 500 *g* for 10 min. The supernatant, which contained the ATP, was recovered in a 1.5 mL centrifuge tube. ATP was quantified using the luciferase-based BacTiter-Glo™ Microbial Cell Viability Assay (Promega, USA). Measurements for the ATP were carried out in a 0.12 M sodium phosphate buffer. To avoid matrix effects potentially caused by the dissolved soil salts a 5-step standard addition was applied^64^. 1, 2, 3, 4 µL of 0.1 µM ATP were added to obtain 100 µL sample aliquots. Finally, 100 µL of BacTiter-Glo™ reagent, which was prepared on the same day, was mixed to the 100 µL of sample solution, blank, or standard. 5 min after mixing, luminescence was recorded using a Glomax 20/20 luminometer (Promega, USA).

### Chlorophyll-a analysis

0.5 g of dry and grounded sample material was extracted with methanol (MeOH) and centrifuged at 2500 U/min for 10 min. After removing the supernatant, the sample material was re-extracted three times and the organic solvents subsequently combined. In order to remove salts, the extract was mixed with dichloromethane (DCM) and water (H_2_O) in a ratio of MeOH/DCM/H_2_O 1:1:0.9 (v/v)^65^ resulting in an organic and aqueous phase. The phases were separated and the aqueous phase was re-extracted using DCM three times. After combining the organic fractions, they were concentrated to dryness using a Turbo Vap 500 and finally a gentle stream of nitrogen. For the sample analysis the extract was solved in 100 µL MeOH. Chlorophyll-a and its degradation products were analyzed using a Thermo Scientific Ultimate 3000 RS ultra-high performance liquid chromatograph (UHPLC) coupled to a Q Exactive Plus Orbitrap mass spectrometer (MS) with a heated electrospray probe (H-ESI II). Samples were separated using a Hypersil gold C18 column (2.1 × 50 mm, 1.9 μm; Thermo Fisher Scientific, Germany) at a temperature of 25 °C. The eluents used for compound separation were (A) methanol and (B) acetonitrile. The solvent gradient was as follows: between 0 and 0.75 min increase of (B) from 10 to 40%, followed by an increase to 50% (B) between 0.75 and 1.5 min, constant at 50% (B) between 1.5 and 5 min and back to 10% (B) between 5 and 6 min which was held constant for further 4 min. The flow rate was set to 0.200 mL/min and an injection volume of 5 µL was used. Electrospray ionization (ESI) source conditions were as follows: spray voltage 3.5 kV; capillary temperature 300 °C; nitrogen sheath gas at 15 and auxiliary gas at 5 arbitrary units at a temperature of 200 °C, the S-Lens was set to 60 V. Automatic gain control (AGC) was used to fill the C-trap and increase the accuracy for mass measurements (AGC target 1 × 106 ions) the maximum IT was set to 50 ms, the number of micro-scans to be performed was set to one. Mass spectra were acquired in positive ion mode at a range of m/z 500 to 1000. The full scan spectra were collected at a high resolution of 280 000 (at m/z 200). Samples have been measured in triplicates and quantified relative to an external chlorophyll a standard (Sigma Aldrich). Chromeleon software was used to process and interpret the data. Chlorophyll-a and its degradation products were identified according to their exact molecular masses (M + H)^+^: chlorophyll-a m/z 893.54259, pheophytin-a m/z 871.57320, pyropheophytin-a m/z 813.56772.

To measure phytol, as another degradation product of chlorophyll-a, an aliquot of the extract was derivatized with N-methyl-N-(trimethylsilyl)trifluoroactetamide (MSTFA) to transform hydroxyl groups into their trimethylsilyl-ether derivatives. Subsequently, the derivatized extract was measured using a Trace Gas Chromatograph 1310 (GC) coupled to a TSQ 9000 Mass Spectrometer (MS) both from Thermo Scientific. The GC was equipped with a cold-injection system operating in the splitless mode and a SGE BPX 5 fused-silica column (50 m length, 0.22 mm inner diameter, 0.25 μm film thickness) using the following temperature conditions: initial temperature 50 °C (1 min isothermal), heating rate 3 °C/min to 310 °C, held isothermally for 30 min. Helium was used as carrier gas with a constant flow of 1 mL/min. The injector temperature was programmed from 50 °C to 300 °C at a rate of 10 °C/s. The MS was operated in the electron impact mode at 70 eV. Full-scan mass spectra were recorded from m/z 50 to 600 at a scan rate of 2.5 scans/s. Phytol was quantified relative to an internal standard (5a-Androstan-17-one, Steraloids Inc.) of known concentration. Due to the high reproducibility of GC-MS data the samples were measured only once.

### Profiling organic matter via FT-ICR-MS

The same extraction and analytical protocol as for similar studies in the region were used to gain comparability^29,66^. Mass spectra were acquired in negative ESI mode using a SolariX Qe FT-ICR-MS equipped with a 12 T superconducting magnet and coupled to an Apollo II ESI-source (Bruker Daltonics, Germany). Methanolic soil extracts were continuously infused with a flow rate of 120 µL/h. Spectra accumulated 300 scans within a mass range of 147 to 1000 m/z. An internal calibration was performed with a mass accuracy of < 0.1 ppm, and peaks with a signal to noise ratio > 6 were picked. Formula assignment was performed with in-house written software (NetCalc) using a network approach to calculate chemical compositions containing carbon, hydrogen, and oxygen, as well as nitrogen and/or sulfur^67^ The mass accuracy window for the formula assignment was set to ± 0.5 ppm, and the assigned formulas were validated by setting sensible chemical constraints (N rule; O/C ratio ≥ 1; H/C ratio ≤ 2n + 2 [maximum possible carbon saturation, with n defined as C_n_H_*n*+2_ for any formula], double bond equivalents) in conjunction with isotope pattern comparison. Results were visualized using van Krevelen diagrams in which the hydrogen to carbon ratio (H/C) was plotted against the oxygen to carbon ratio (O/C). The different bubble sizes represent the intensity of the characteristic molecular formula within the respective sample.

## Supplementary Information

Below is the link to the electronic supplementary material.

Supplementary Material 1

## Data Availability

All data are provided within the manuscript, the supplementary information files, or in the DepositOnce repository (DOI: https://doi.org/10.14279/depositonce-23322). The FT-ICR-MS raw data are available upon request from the corresponding author and can be adapted depending on the intended use.
