# Supplementary material for: Exploring organic compound preservation through long-term in situ experiments in the Atacama desert and the relevance for Mars
Source: Sci Rep. 2025 Aug 15;15:29957. doi: 10.1038/s41598-025-16197-w (PMC12356877; doi:10.1038/s41598-025-16197-w)
Supplement: Supplementary file 1 — Supplementary Material 1 [file 41598_2025_16197_MOESM1_ESM.pdf]

Supplementary Information

for

Exploring organic compound preservation through long-term in situ experiments in the Atacama Desert and their relevance to Mars

Environmental monitoring results

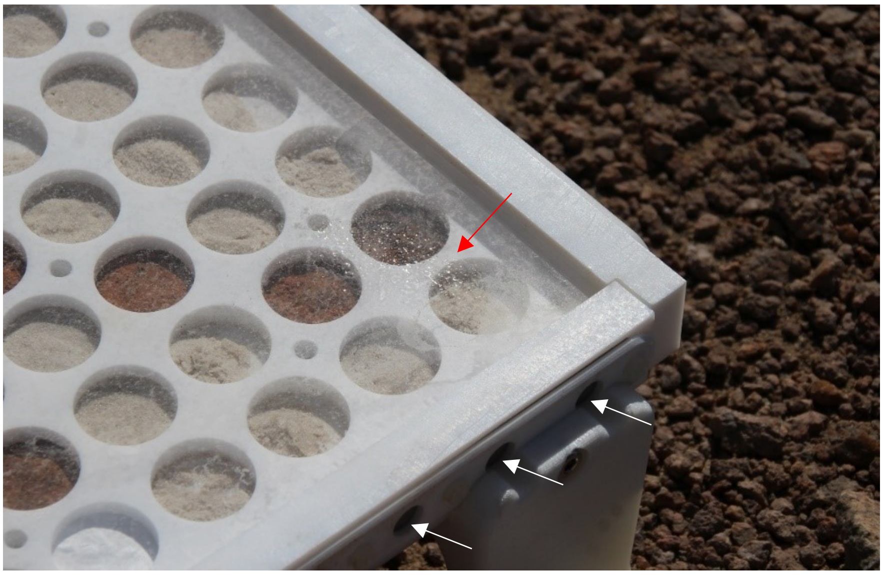

Figure S 1 Dew formation (red arrow) in the sample plates below the glass cover on the 31st of January 2023. Air vents (white arrows) are located on the side of the plate.

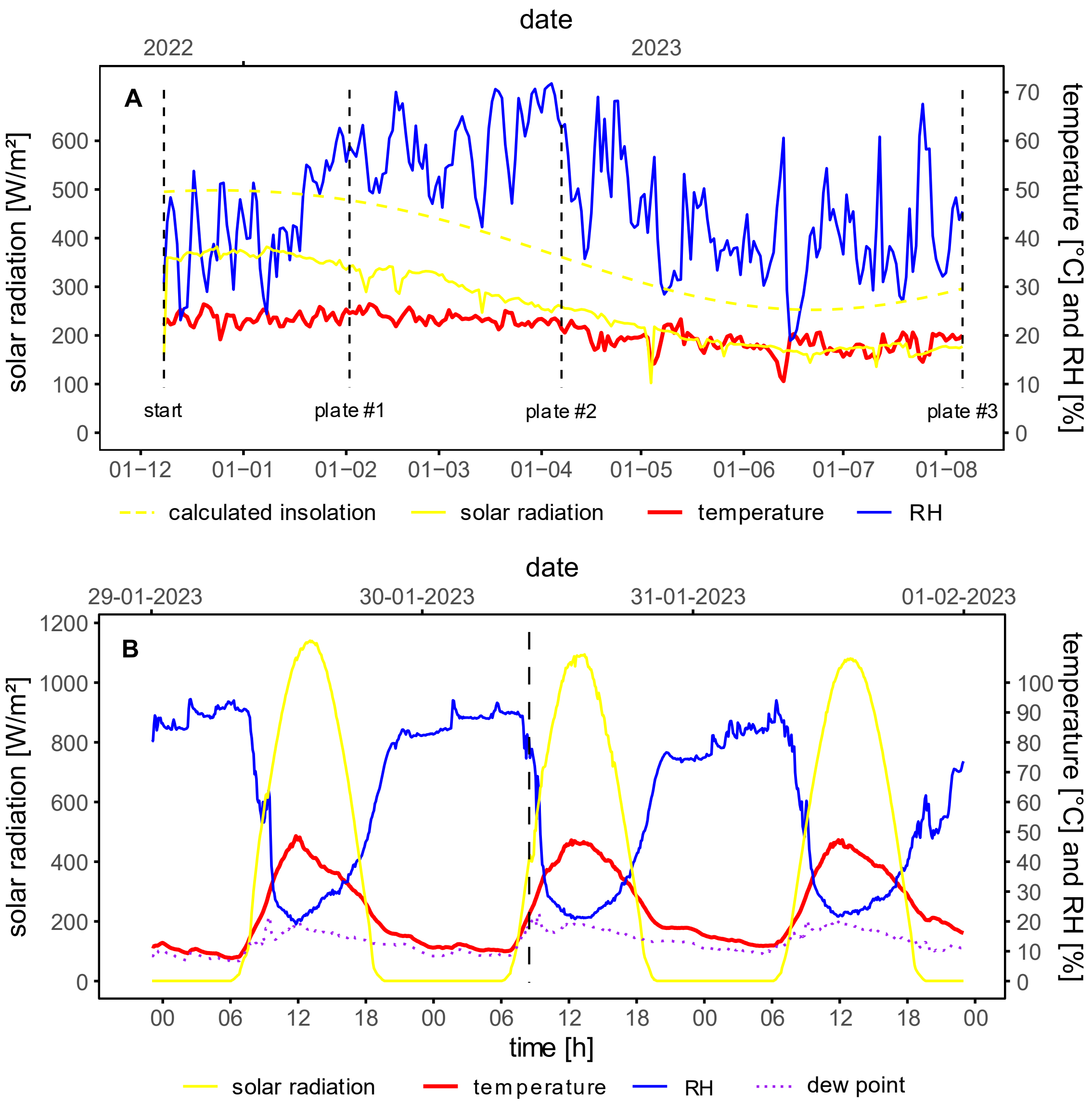

Figure S 2 Environmental monitoring data. A) Over the duration of the field experiment with daily average values of temperature, RH and solar radiation. The experiment started on the 08-12-2022, the plate #1, #2, and #3 were picked up on 02-02-2023, 07-04-2023, and 07-08-2023, respectively. B) Over the duration of three days during (29-01 to 01-02-2023) the field experiment, including the calculated dew point (in °C). Record interval is 5 minutes. During the morning of the 31-01-2023 (dashed line) we observed dew on the experiment setup.

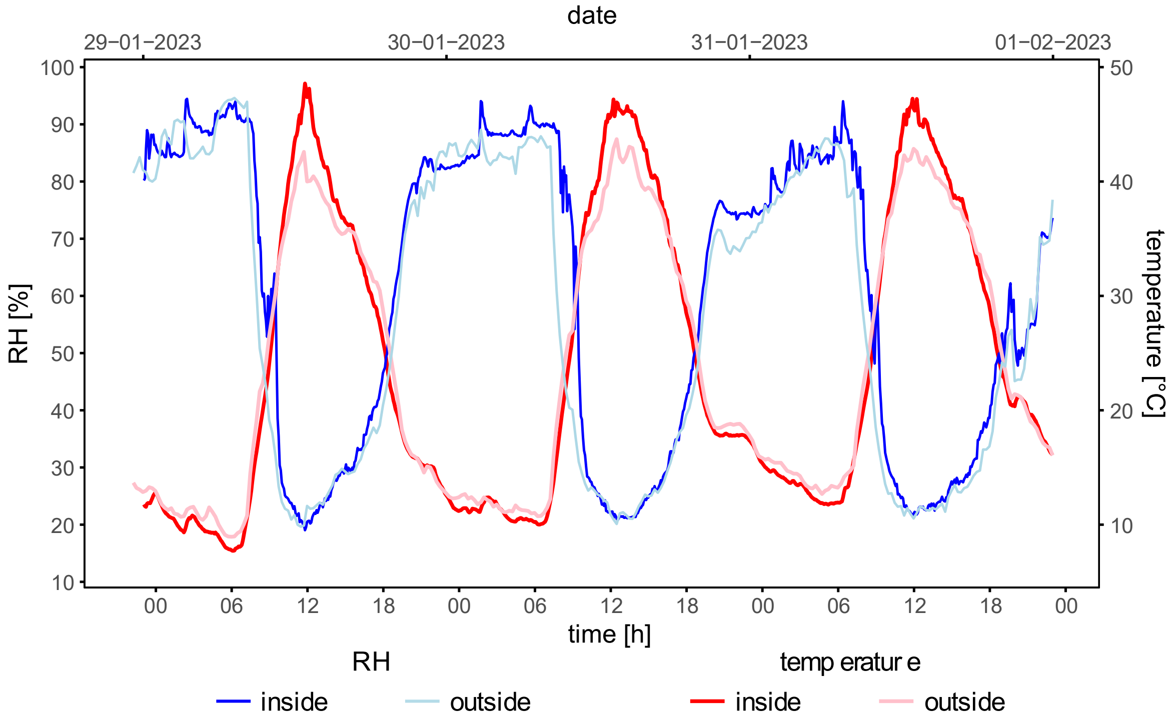

Figure S 3 Comparison of the temperature and RH inside and outside (surface) of the sample plates. Same time frame used as in Figure S 2B.

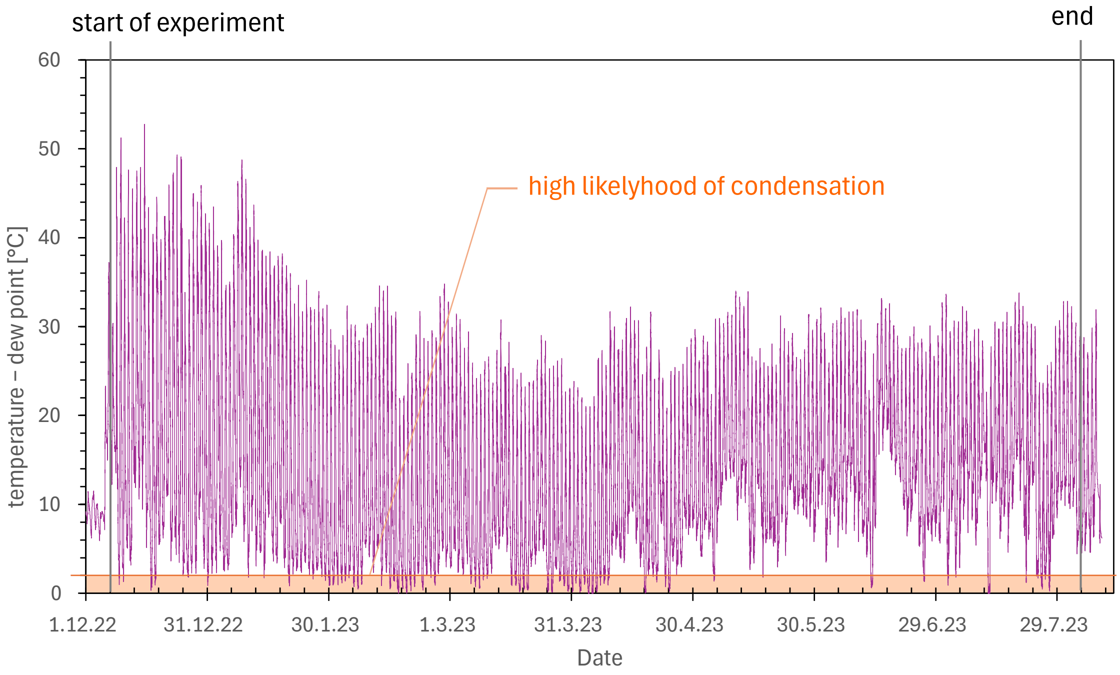

Figure S 4 Periods of high likelihood of condensation within the sample plates over the course of the experiment. Values were calculated as the difference between dew point and temperature (temperature – dew point [°C]). A difference of ≤2 °C indicates a high likelihood of condensation. In total, potential condensation occurred for approximately 320 hours (~5% of the experiment period).

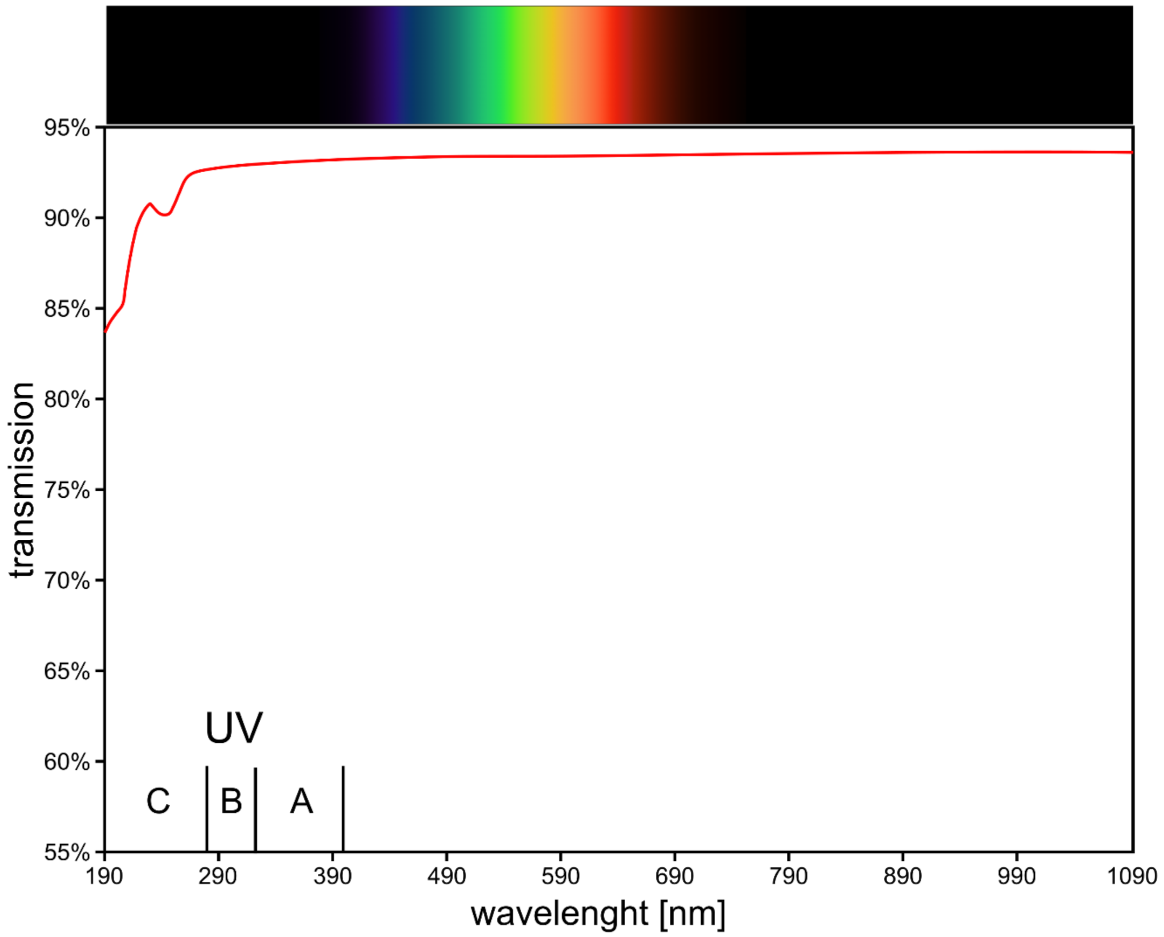

Figure S 5 Transmission spectrum of the quartz glass (FN08) used for the plate cover during the experiment (source GVB GmbH).

Table S 1 MGS-1 composition and bulk chemistry supplied by exolith lab batch no. 002-05-001-0621 which was used for the experiment.

**Component**	**Wt%**	**Oxide**	**Wt%**
Anorthosite	27.1	SiO2	42.9
Glass-rich basalt	22.9	TiO2	0.6
Pyroxene	20.3	Al2O3	12.8
Olivine	13.7	FeO	11.2
Mg-sulfate	4.0	MnO	0.1
Ferrihydrite	3.5	MgO	14.6
Hydrated silica	3.0	CaO	7.4
Magnetite	1.9	Na1O	1.5
Fe-carbonate	1.4	K2O	0.6
Hematite	0.5	P2O5	0.1
		Loss on ignition	5.3
		Total*	97.1

*excluding volatiles and trace elements

Table S 2 ATP concentration in mol/g of the exposure experiment after different exposure durations. The standard error is derived from the standard addition calibration.

**sample**	**ATP [mol/g]**
quartz C (no exposure)	56.7±1.8 10^{-9}
quartz 2 months exposure	31.0±2.4 10^{-9}
quartz 4 months exposure	6.8±1.9 10^{-9}
quartz 8 months exposure	3.7±1.1 10^{-9}
MGS-1 C (no exposure)	319.0±166.0 10^{-9}
MGS-1 2 months exposure	26.6±1.3 10^{-9}
MGS-1 4 months exposure	6.1±0.9 10^{-9}
MGS-1 8 months exposure	11.2±3.0 10^{-9}
gypsum C (no exposure)	255.0±53.5 10^{-9}
gypsum 2 months exposure	267.0±95.9 10^{-9}
gypsum 4 months exposure	137.0±3.5 10^{-9}
gypsum 8 months exposure	357.0±66.5 10^{-9}
quartz NaCl C (no exposure)	59.6±2.8 10^{-9}
quartz NaCl 2 months exposure	11.8±2.5 10^{-9}
quartz NaCl 4 months exposure	1.7±4.2 10^{-9}
quartz NaCl 8 months exposure	2.5±0.8 10^{-9}
quartz $NaClO_4$ C (no exposure)	94.1±5.8 10^{-9}
quartz $NaClO_4$ 2 months exposure	36.7±0.4 10^{-9}
quartz $NaClO_4$ 4 months exposure	8.7±1.4 10^{-9}
quartz $NaClO_4$ 8 months exposure	9.7±1.3 10^{-9}

Table S 3 Chlorophyll-a and its degradation products in ng/g substrate. The larger molecules detected by LC-MS were measured in triplicates and the values represent an average with standard deviation. Phytol detected by GC-MS was only measured once. n.d. = no detection, n.a. = not analyzed, C = control sample.

**sample**	**chlorophyll-a**	**pheophytin-a**	**pyropheophytin-a**	**phytol**
quartz C (no exposure)	n.d.	375.87±8.10	34.42±3.21	22.81
quartz 2 months	n.d.	n.d.	n.d.	14.01
quartz 4 months	n.d.	n.d.	n.d.	n.d.
quartz 8 months	n.d.	n.d.	n.d.	n.d.
quartz NaCl C (no exposure)	n.d.	99.39±6.93	4.38±0.60	46.48
quartz NaCl 2 months	n.d.	n.d.	n.d.	11.5
quartz NaCl 4 months	n.d.	n.d.	n.d.	n.d.
quartz NaCl 8 months	n.d.	n.d.	n.d.	n.d.
quartz $NaClO_4$ C (no exposure)	n.d.	9.87±0.47	0.26±0.04	132.91
quartz $NaClO_4$ 2 months	n.d.	n.d.	n.d.	n.a.
quartz $NaClO_4$ 4 months	n.d.	n.d.	n.d.	n.d.
quartz $NaClO_4$ 8 months	n.d.	n.d.	n.d.	n.d.
gypsum C (no exposure)	n.d.	12.22±0.59	48.98±5.70	976.79
gypsum 2 months	n.d.	n.d.	n.d.	13.59
gypsum 4 months	n.d.	n.d.	n.d.	n.d.
gypsum 8 months	n.d.	n.d.	n.d.	n.d.
MGS-1 C (no exposure)	n.d.	9.78±0.60	72.73±4.62	1448.73
MGS-1 2 months	n.d.	0.57±0.17	1.05±0.04	56.24
MGS-1 4 months	n.d.	0.50±0.05	0.35±0.01	4.84
MGS-1 8 months	n.d.	0.10±0.02	0.03±0.01	4.78
